# The Childhood Trauma Questionnaire—Short Form (CTQ-SF) used with adolescents – methodological report from clinical and community samples

**DOI:** 10.1007/s40653-022-00443-8

**Published:** 2022-03-30

**Authors:** Johan Melander Hagborg, Torbjörn Kalin, Arne Gerdner

**Affiliations:** 1 Department of Psychology, Gothenburg University, Gothenburg, Sweden; 2 School of Health and Welfare, Department of Social Work, Jönköping University, Jönköping, Sweden

**Keywords:** Childhood maltreatment, Adolescent screening, Acceptability, Reliability, Validity, Norm data

## Abstract

**Purpose:**

The Childhood Trauma Questionnaire—Short Form (CTQ-SF) is a widely used retrospective screening tool for childhood maltreatment in adults. Its properties are less known in adolescents. The objective was to investigate acceptability and psychometric properties when used in adolescents.

**Method:**

A community sample of adolescents (n=1885) in four waves (from 13 or 14 to 17 years old) and a clinical sample (n=74, mean age 18), both from Sweden, were used to assess acceptability and different aspects of validity and reliability.

**Results:**

The CTQ-SF was found to be well-accepted. As expected, the community sample scored lower than the clinical sample on all maltreatment-scales and showed stability over-time. In the community sample, internal consistencies were substantial or excellent for all scales except Physical neglect, and in the clinical sample this was found for all scales. One-year test-retest consistencies of subscales were substantial or almost perfect, and for all scales, they increased from early to mid-adolescence. Directed inconsistencies on item level decreased from early to mid-adolescence. Convergent validity was shown in relation to scales on family climate, parental relations, and emotional health also from early adolescence. Discriminant analyses showed more moderate discriminatory ability although almost seven times better than by-chance.

**Conclusions:**

The CTQ is well accepted and can be trusted to provide consistent and valid self-reports from the age of 14 on childhood maltreatment. Some caution is advised when used with younger adolescents, since the test-retest stability is then weaker, and the interpretation of the M/D scale is more ambiguous.

## Introduction

Child maltreatment exemplifies a harmful relational environment that poses significant risks for maladaptation across biological, social, and psychological domains of development from childhood to adulthood (Cicchetti & Toth, 2005). Child maltreatment is usually divided into two subcategories: actions of omission (emotional and physical neglect) and acts of commission (physical, emotional, and sexual abuse; Barnett et al., 1993). The World Health Organization (WHO) distinguishes four types of child maltreatment: physical abuse, sexual abuse, emotional or psychological abuse, and neglect (Butchart et al., 2006; WHO, 2020). Worldwide, 23% of adults retrospectively report physical abuse, 36% emotional abuse, and 16% physical neglect, while 18% of women and 8% of men report sexual abuse in their childhood (WHO, 2020). In Sweden, results from recent studies on adolescent populations suggest that 13% retrospectively report physical abuse, 8-13% report emotional abuse and 14% of girls and 3-5% of boys report sexual abuse (Hagborg et al., 2018; Jernbro & Jansson, 2017).

In a review of research on the developmentally salient outcomes of child maltreatment during adolescence, Trickett et al. (2011) found extensive evidence for the impact of child maltreatment on adolescent development. For example, early maltreatment has been shown to have a negative impact on affect regulation, attachment relationship formation, and self-system development (Cicchetti & Rogosch, 2002; Trickett et al., 2011). This may lead to an impaired ability to become more self-directed and independent and to manage close relationships outside the immediate family (Cicchetti & Rogosch, 2002). Maltreated adolescents therefore tend to have more problems in peer relationships, romantic relationships, and academic functioning than their non-maltreated peers (Trickett et al., 2011). Delinquency and substance use have also been found to be more common among maltreated adolescents (Trickett et al., 2011). A remaining impact in adulthood is suggested by associations with such as epigenetics (Bendre, 2019), the serotonergic system (Berglund et al., 2013), and the immune defence system (Carlsson, 2016).

There are different ways of gathering data about child maltreatment. Methods typically involve retrospective self-reports from adults, reports from caregivers, observations of caregiver behaviour, and/or analyses of records from child welfare services and/or medical records. These different approaches yield to very different prevalence rates (Shaffer et al., 2008). For example, given that only a small number of maltreatment cases are identified through child protective services (CPS) reports, relying exclusively on such reports risks underestimating the incidence of maltreatment in a population (Briere 1992; Kendall-Tackett & Becker-Blease, 2004). Self-reports, however, can be influenced by subjective interpretations of questionnaire items and/or different degrees of willingness to report acts of child maltreatment (Weeks & Widom, 1998; Widom et al., 2004). Shaffer et al., (2008) found that the cases with the greatest number of incidences of maltreatment were likely to be identified by both retrospective self-report and official records. In a new study, Kalin et al. (2021) found that many of the most severe cases were in fact not detected by child welfare services. Hence, it is important to identify reliable self-report measures that can correctly evaluate to what degree adolescents have been exposed to child maltreatment.

From a critical review, Hardt and Rutter (2004) examined the evidence of validity of retrospective reports by adults of their own adverse experiences in childhood and found a substantial rate of false negatives and measurement errors, but also that false positive reports were probably rare. They argued that comparison of contemporaneous and retrospective accounts obtained in epidemiological/longitudinal studies of non-clinical populations is the best method to address this issue of validity. A similar approach, using long-term repeated measures in longitudinal studies, was used by Fan et al. (2006) to identify incorrect responders in adolescent self-reports.

Among self-report measures, the short form of the Childhood Trauma Questionnaire, (CTQ-SF; Bernstein & Fink, 1998; Bernstein et al., 2003) is one of the most widely used scales and has been translated into more than a dozen languages (Daalder & Bogaerts, 2011; Garrusi & Nakhaee, 2009; Grassi-Oliveira et al., 2006; Paquette et al., 2004; Thombs et al., 2009; Wingenfeld et al., 2010, see also MacDonald et al. 2016).

The psychometric properties of the Swedish version of the CTQ-SF have been explored in both clinical and non-clinical adult samples giving retrospective reports on their childhood (Gerdner & Allgulander, 2009). The non-clinical samples were university students, not epidemiologic community samples. Epidemiological norm data from non-clinical adolescent populations in Sweden are still lacking. The psychometric properties of CTQ-SF when used in adolescent community samples are also less known, as its acceptability for use among adolescents (Nilsson & Svedin, 2017).

Even in a severely traumatised clinical sample of addicted women with psychiatric comorbidity, the CTQ-SF was found to be acceptable and non-intrusive (Lundgren et al., 2002). Epidemiological studies on adolescents using other instruments report that 4-15% felt upset by questions on emotional problems, including questions on childhood trauma (Finkelhor et al., 2014; Hasking et al., 2015). However, we saw no study reporting specifically on adolescent acceptability of the CTQ.

Bernstein & Fink (1998) published norm data from an adolescent clinical population (n=398, aged 12-17), but not from an adolescent community population. Swedish norm data on adolescent populations are still lacking. Combining clinical and non-clinical adolescent samples in the same study makes it possible to study discriminant ability, i.e., the ability of the CTQ to discriminate between the clinical and non-clinical samples.

The test-retest consistency of the CTQ was studied by the creators in a clinical population (n=40) showing substantial test-retest reliability with retests conducted after 1.6-5.6 months (mean 3.6 months) and showing a high intraclass correlation of all subscales (R=.79–0.86) (Bernstein & Fink, 1998). Test-retest was also tried in three independent studies in different populations (Cammack et al., 2016; Kim et al., 2013; Paivio, 2001). Cammack and colleagues studied test-retest during pregnancy and after completion of the pregnancy with a substantial proportion of women who reported exposure to maltreatment. They found test-retest reliability to be at least moderate, indicating consistent reporting. Kim and colleagues studied outpatient and inpatient schizophrenia patients and found high test-retest reliability. Paivio conducted test-retest of the CTQ before and after therapy of maltreatment victims. Although there is moderate to high consistency in all these studies, which involved various populations of adults, the nature of inconsistencies should also be analysed. Decline in recall accuracy and detail is one proposed factor and could increase with a greater time lapse between the events and the survey (e.g., Williams, 1994). Embarrassment or a wish to protect the perpetrator may result in conscientious underreports (Melchert & Parker, 1997). Positive reconstructing (minimisation) is another factor, resulting in under-reports of maltreatment being more frequent than over-reports (Della Femina et al., 1990; Fergusson et al., 2000).

Test-retest data concerning the CTQ-SF in adolescent populations are scarce but should contribute to the understanding of the ecological validity of the CTQ-SF. Adolescents report their experiences closer in time and could therefore have less memory bias. Lack of distance to the experiences at very young age might also be a problem, if the most recent events are given more attention than the general situation. All the proposed factors involved in explaining inconsistencies could be influenced by age at the time of reporting. Specifically addressing the test-retest stability of self-report measures within the developmental period of adolescence could give important information about the nature of reporting bias in retrospective self-report measures of child maltreatment (Hardt and Rutter, 2004). It is therefore relevant to examine whether the CTQ works similarly when used in an adolescent population, and from what age it can best be used.

Another issue concerns the M/D scale in the CTQ, which is used to identify people where there is a risk of minimisation and denial. Unrealistic extreme scorings on the three M/D items are assumed to demonstrate such risks, and the clinician is encouraged in such cases to try to find additional data sources that can confirm the assessment (Bernstein & Fink, 1998). This way of interpreting M/D had support in the Swedish study (Gerdner & Allgulander, 2009) which showed that high M/D is consistent with the propensity to give socially desirable rather than correct answers. Further support for the position was given in a large collaborative international study on the M/D scale by MacDonald and others (2016). A related question is therefore whether the interpretation of M/D should be the same when used in an adolescent population.

The CTQ-SF is recommended to be used from the age of twelve (Bernstein & Fink, 1998), based on its use in the adolescent clinical population aged 12–17 (Bernstein et al., 1997). There is, however, a lack of studies evaluating its use in early adolescence based on analyses of its acceptability, consistency and functional equivalence of items and scales, including M/D. Prior studies on adolescents mainly include children aged 15 and older, mainly addressing high-risk populations (Aloba et al., 2020; Charak et al., 2017, Dovran et al., 2013; Grassi-Oliveira et al., 2014), and to our knowledge this is the first study on adolescents addressing test-retest.

In conclusion, there are extensive psychometric studies internationally regarding the use of the CTQ-SF in adults, but there are fewer studies concerning adolescents both in community and in clinical populations. No equivalent study on adolescents in a non-clinical community sample was found. It is important to test its acceptability as well as its validity and reliability for adolescents, publish normative data, and address the issue of how the Minimising/Denial scale works in adolescent populations.

The aims are to present acceptability and psychometric data on the CTQ-SF when it is used repeatedly in a community adolescent population. This includes means and distributions over the years, intercorrelations between scales and between waves, internal consistencies over the years, global (test-retest) consistencies on both scale and item levels, also testing directionality in the case of non-consistent items, convergent validity, and norm data in adolescents. Based on that, a further aim is to evaluate from which age the CTQ-SF can be recommended, and then if the M/D scale can be used in the same way. Additional aims are to present psychometric data in an adolescent clinical sample and the ability of the CTQ-SF to discriminate between the community and clinical samples.

## Methods

### The instrument

Childhood Trauma Questionnaire–Short Form (CTQ-SF; Bernstein & Fink, 1998) consists of 28 items, of which 25 measure childhood maltreatment (total), including five subscales of five items each, i.e., Emotional Abuse (EA), Physical Abuse (PA), Sexual Abuse (SA), Emotional Neglect (EN) and Physical Neglect (PN). Three items are designed to measure Minimisation/Denial (M/D). All 28 items are constructed as statements beginning with the phrase ‘When I was growing up…’ The key wordings of the items that followed are shown in Table 4. All five abuse and neglect subscales are sums of the scorings from ‘never true’ (score 1) to ‘very often true’ (score 5), and after reversing seven items, all subscales can therefore vary between 5 and 25. The M/D scale is different, since only the highest positive scores (score 5) are counted, and it can therefore vary from 0 to 3. Ratings of statements, such as ‘I had the perfect childhood’ with the highest possible score, are unrealistic and therefore biased. Positive scores on M/D may therefore be problematic, and it has been recommended in such cases to interpret clinical assessment on maltreatment scales with caution and possibly try to validate data through other sources (Bernstein & Fink, 1998; Gerdner & Allgulander, 2009; MacDonald et al., 2016). The same items can also be used as a summative scale in full length (3-15) as suggested to measure Idealising Upbringing (IU), and not necessarily a bias (Gerdner & Allgulander, 2009).

### Data collections

#### Community sample

Data from the LoRDIA programme was used. LoRDIA (Longitudinal Research on Development In Adolescence) followed adolescents of two year-cohorts in four small and medium-sized municipalities (10,000-38,000 inhabitants) in the south of Sweden. Two municipalities are industrial and two are commuting communities, linked to nearby cities. One of the commuter municipalities is located near a large city. The unemployment rate, annual income, educational level, and proportion of first-generation immigrants across the four municipalities were close to the national means (Statistics Sweden, 2019).

In 2013, all adolescents in grades 6 (cohort A; 12 years old) and 7 (cohort B; 13 years old) were invited to take part in the programme. Annual data collections were then conducted. Cohort A had data collections in grade 6, 7, 8 and 9. Cohort B had data collections in grades 7, 8 and 9 Both cohorts had an additional data collection when they were in the second year of high school, aged 17.

All parents had been informed by letter (to both parents if they lived separately) about the aims and scope of the programme, its longitudinal character, and their right to decline participation on behalf of their child. The letters had been translated into the home language (32 different languages other than Swedish). If the parents did not decline their child’s participation, the child was invited and given the same information in content, although adapted in form to their age. They were then informed of their right to decide for themselves whether to take part, including their right to opt out. Of all 2150 invited by parent letter, 1885 students (88%) finally remained in the programme. Those who opted out (193 on the parent’s decision, 73 due to their own decision) did not differ from the study population in terms of gender, immigrant status (studying Swedish as second language), school merits or absenteeism when compared to participants using school register data. Each data collection wave of the research programme was approved by the Ethics Review Board in Gothenburg (Nr. 362-13; 2013-09-25; 2014-05-20; 2015-07-31; 2017-07-21).

The CTQ was used repeatedly in four waves, in part starting in Wave 2 (aged about 13 and 14 years), and in full starting in Wave 3 (14 and 15 years) and was then repeated in Wave 4 (one cohort, about 15 years), and Wave 5 (about 17 years). In Wave 2, only 13 of the 28 items were used (subscales EN, EA and the three items of M/D). This decision was taken for two reasons, one was that sexual and physical abuse was regarded as very sensitive at such young age, and the other was to save space since the Wave 2 questionnaires included a comprehensive personality inventory. The first three waves (W2-4) had the same time frame, asking the respondents about their childhood situation ‘before you were 12 years old’. In Wave 5, when they had reached the age of 17, the time frame was changed to ‘before you were 15 years old’. The reason for that was the possibility of finding emerging problems with onset during adolescence. The responders were not informed that the questions were to be repeated in new data collections.

#### Clinical sample

The data collection for the clinical sample was conducted in 2016 at nine outpatient clinics located in south and central Sweden. The clinics are specialised units aimed at young people with substance use problems and are run in collaboration between social services and health care. All clinics offer various forms of family therapeutic treatment and manual-based treatment programs for alcohol and drug addiction, often with multimodal approaches. The average length of care is four to six months (Anderberg & Dahlberg, 2018).

At the beginning of treatment, the staff carry out a survey based on the structured interview method UngDOK (Dahlberg et al., 2017). For this study, a number of participants were asked if they also wanted to complete a self-assessment form about experiences of child maltreatment before the age of 12, i.e., the CTQ-SF. In these matters, a short screening questionnaire is preferable to an interview since it is less intrusive and gives more valid replies (Kim et al. 2008). A total of 74 young people (39% girls) chose to fill out the CTQ-SF. The average age was 18 years with a spread of 14-25 years. The study was approved by the Ethics Review Board in Gothenburg (Dnr. 2015/160-31).

### Analytical design

The data obtained were used for several analyses and reports:

1. To determine the acceptability of the CTQ based on community respondents´ comments.
2. For both the clinical and community samples (all waves), the distributions of childhood problems are presented.
3. Intercorrelations between scales and between waves in the community sample were tested using Pearson R. Same-scale correlations were also tested separately for those with and without M/D=0.
4. The internal inter-item consistencies of all scales (Cronbach’s alpha) are presented for both samples and for all waves in the community sample.
5. Long-term consistency in one-year test-retests were examined in the community sample both on item and scale levels by comparing waves 2 and 3, as well as waves 3 and 4. Since all items were ordinal, the method to test agreement was the symmetric Gamma (γ). Gamma estimates systematic agreement when corrected for random agreement, varying from 1 in the case of perfect agreement, to 0 which is not more than random agreement (Goodman & Kruskal, 1954). Negative values exist when agreement is less than random. Gamma is the equivalent of Cohen’s Kappa applied to ordinal scales and can be interpreted in the same way, i.e., as follows: <0.00 ‘poor’, 0.00-0.20 ‘slight’, 0.21-0.40 ‘fair’, 0.41-0.60 ‘moderate’, 0.61-0.80 ‘substantial’, and >0.81 is ‘almost perfect’ (Landis & Koch, 1977; Gerdner & Wickström, 2015).
6. Inconsistencies were further analysed for possible directionality in over- versus under-reporting, i.e., whether adolescents tended to change their evaluation of childhood in more positive or in more negative ways, using the Wilcoxon Sign Rank test (Gibbons, 1993).
7. Convergent validity was examined in the community sample in three waves, by same-wave correlations to scales chosen to reflect family climate, parental relations, and the child´s emotional health. For the clinical sample, convergent validity was examined in relation to emotional health.
8. Discriminant validity, i.e., the CTQ´s ability to discriminate between the samples, was tested.
9. Norm data: The percentiles of the subscales are presented for early and mid-adolescence (waves 3 and 5) in the community sample and for late adolescence in the clinical sample.

### Measures for testing convergent validity

Convergent validity is tested against relevant scales from waves 2, 3 and 5 (W2, W3 and W5) of the community sample and from the clinical sample, presented with the respective internal consistency (Cronbach’s alpha, α).

Measures of family climate: *Family cohesion* and *Family conflict* are two scales from Bloom (1985), each consisting of five questions. Example of cohesion: ‘In our family, we help and support each other’ (α[W2, W5]=0.70 and 0.79); example of conflict: ‘In our family we fight a lot with each other’ (α[W2, W5]=0.63 and 0.66). *Witnessed domestic violence* is a question added in the format of the CTQ: ‘When I was growing up, I witnessed violence between adults in my home’.

Measures of parental relations: *Perceived maternal support* and *Perceived paternal support* (Tilton-Weaver, 2014) are measured separately, each based on five questions, e.g. ‘I know mom/dad is there when I need her/him’ (α[W2]=0.87 and 0.88). *Parental substance misuse* is based on a question asked about adults in the family, independent of biological relations: ‘An adult in the family I lived with–parent, step-parent or other–had severe alcohol or drug problems.’

Measures of emotional health: *Psychological Health* (Gustavsson et al., 2012; Hagquist, 2005) measures positive health from three items, e.g. ‘How do you in general enjoy life just now’ (α[W2-W3, W5]=0.76, and 0.72). *Mental Health Continuum–Short Form* (MHC-SF; Lamers, et al., 2011), measures satisfaction with life using 14 items, all starting with ‘How often in the last month have you felt’ followed by e.g., ‘happiness, joy’, ‘an interest in life’, ‘that people are mainly good’ (α clinical=0.92; community W3=0.95; W5=0.94). From the Strength and Difficulties Questionnaire (SDQ, Goodman, 1997; Smedje et al., 1999) we used two subscales. One was *Emotional problems*, with five questions, e.g. ‘I often have headaches, stomach aches, or nausea’ (α[W2-W3, W5]=0.77, 0.75, and 0.72). Another was the *Impact of problems, e.g.* ‘Are you bothered or troubled by the difficulties?’ α[W2-W3, W5]=0.81, 0.76, and 0.78).

## Results

### Acceptability

When we first introduced the CTQ in the study, we hesitated to use the full instrument, especially physical and sexual abuse, with 13-year-olds. In Wave 2, CTQ therefore was only used in part. When using it fully in waves 3-5 however, we never received any critical remarks on these questions, according to protocols from the data collections. In addition, the questionnaires ended with questions concerning how it felt to fill in the questionnaire. In Wave 3 with 390 questions, 88% stated that they felt the questions overall were important, and 98% stated that they had replied honestly to all. They were also given the possibility to comment on specific questions. Of all 1321 respondents, 200 chose to make some comment. Only three (0.2% of responders) commented on the CTQ, and of these, two were critical of questions on sexual abuse, and one stated that the questions were too personal and abstained from answering. In waves 4 and 5, ratings were almost identical, but the only comment on the CTQ in Wave 4 was that the respondent would also have liked to have questions on parental physical abuse of siblings, and in Wave 5 none commented on the CTQ. We conclude that the CTQ in general was well accepted.

### Outcomes in community samples (four waves) and clinical sample

Table 1 presents outcomes on global childhood maltreatment (CTQ total) and the different subscales, separately for boys and girls in four waves over the years from the community sample, as well as for boys and girls in the clinical sample.

**Table 1:** *Outcomes of the CTQ and its subscales (means, SD) in Swedish adolescent boys and girls in four waves of the community sample, as well as in a clinical sample of Swedish adolescents undergoing outpatient substance misuse treatment*

		Adolescents, community population				Adolescents clinical	
	Wave 2 (grades 7 & 8) Boys, 13+ yrs		Wave 3 (grades 8 & 9) Boys, 14+ yrs	Wave 4 (grade 9) Boys, 15 yrs	Wave 5 (high school grade 2) Boys, 17 yrs		Boys 14-25 yrs 18 yrs (mean)
n =	689		645	365	415		45
CTQ total			31.4 (9.43)	31.6 (9.60)	31.0 (8.10)		37.2 (11.94)
EN	7.7 (3.21)		7.3 (3.32)	7.4 (3.42)	7.5 (3.45)		9.6 (4.69)
PN			6.4 (2.30)	6.7 (2.50)	6.3 (2.19)		7.6 (3.70)
EA	7.4 (3.16)		6.8 (2.78)	6.7 (2.68)	6.6 (2.57)		8.0 (3.81)
PA			5.6 (2.05)	5.6 (2.03)	5.3 (1.57)		5.6 (1.57)
SA			5.3 (1.81)	5.2 (1.54)	5.2 (1.33)		5.7 (2.72)
M/D	1.1 (1.03)		1.2 (1.15)	1.3 (1.19)	1.2 (1.16)		0.7 (0.93)
IU (a)	11.1 (2.43)		11.4 (2.78)	11.5 (2.75)	11.5 (2.82)		-
	Wave 2 (grades 7 & 8) Girls, 13+ yrs		Wave 3 (grades 8 & 9) Girls, 14+ yrs	Wave 4 (grade 9) Girls, 15 yrs	Wave 5 (high school grade 2) Girls, 17 yrs		Girls 14-25 18 yrs (mean)
n =	739		657	346	520		29
CTQ total			30.9 (7.54)	31.4 (8.82)	30.3 (7.12)		43.9 (20.36)
EN	7.4 (3.16)		7.4 (3.29)	7.6 (3.74)	7.1 (3.10)		10.1 (4.54)
PN			6.1 (1.91)	6.2 (1.96)	6.1 (1.92)		8.0 (3.82)
EA	7.0 (2.84)		6.9 (2.81)	7.0 (3.20)	6.7 (2.66)		10.9 (6.14)
PA			5.4 (1.28)	5.4 (1.18)	5.2 (0.87)		6.8 (3.42)
SA			5.1 (0.90)	5.2 (1.60)	5.2 (1.21)		7.4 (5.45)
M/D	1.1 (1.10)		1.1 (1.17)	1.2 (1.21)	1.1 (1.17)		0.7 (1.10)
IU (a)	11.2 (2.67)		11.4 (2.80)	11.4 (3.01)	11.6 (2.83)		-

a) IU could not be calculated for the clinical sample, since only dichotomised replies on the M/D items were provided

CTQ=Childhood Trauma Questionnaire, EN=Emotional Neglect, PN=Physical Neglect, EA=Emotional Abuse, PA=Physical Abuse, SA=Sexual Abuse, M/D=Minimizing/Denial, IU=Idealizing Upbringing

In the community population, the difference in scale means over the waves were small for both boys and girls when measured on group levels, although boys scored somewhat more on EN and EA in Wave 2 than in later waves. Since individual participation differed between waves, mean differences may partly reflect selection. The clinical sample scored higher than the community sample (compared to Wave 5, being most similar in age) on all scales except the M/D scale which was lower.

### Intercorrelations between scales in two waves of the community sample

In Table 2, intercorrelations between CTQ scales were studied both within and between the two waves that had data on all subscales and examined in both age cohorts, i.e., Wave 3 at the age of 14-15 years and Wave 5 at the age of about 17 years. In addition, same-scale correlations were also investigated for groups with and without any positive scores on M/D.

**Table 2:** *Intercorrelations (Pearson R) between the CTQ and its subscales in Wave 3 (above diagonal; n = 1302) and Wave 5 (below diagonal; n = 934) as well as same scales between the two waves (diagonal; n = 733); the latter also separately for groups with and without M/D>0*

	CTQ total	EN	PN	EA	PA	SA	M/D	IU
CTQ total	0.48***	0.81***	0.75***	0.83***	0.69***	0.56***	- 0.38***	- 0.52***
EN	0.81***	0.47***	0.58***	0.57***	0.32***	0.17***	- 0.49***	- 0.63***
PN	0.69***	0.48***	0.27***	0.47***	0.40***	0.28***	- 0.24***	- 0.37***
EA	0.80***	0.54**	0.34***	0.49***	0.50***	0.42***	- 0.32***	- 0.44***
PA	0.60***	0.24***	0.31***	0.47***	0.24***	0.58***	- 0.09**	- 0.17***
SA	0.50***	0.15***	0.26***	0.32***	0.53***	0.32***	- 0.04	- 0.07**
M/D	- 0.41***	- 0.49***	- 0.22***	- 0.32***	- 0.10**	- 0.04	0.60***	0.78***
IU	- 0.60***	- 0.66***	- 0.37***	- 0.47***	- 0.19***	- 0.10**	0.77***	0.59***
Diagonal correlations when cases with *no* extreme values on the M/D scale are included.								
M/D=0 (n=276)	0.52***	0.47***	0.38***	0.53***	0.19***	0.51***		
Diagonal correlations when *cases with some* extreme values on the M/D scale are included.								
M/D>0 (n=457)	0.26***	0.15***	0.13***	0.30***	0.29***	0.18***		

* p < .05; ** p < .01; *** p < .001

CTQ=Childhood Trauma Questionnaire, EN=Emotional Neglect, PN=Physical Neglect, EA=Emotional Abuse, PA=Physical Abuse, SA=Sexual Abuse, M/D=Minimizing/Denial, IU=Idealizing Upbringing

Most maltreatment scales were highly positively correlated (Rs >0.40) and in both waves, except for more moderate (although significant) correlations for EN/SA (Rs [W3;W5]=0.17 and 0.15). PN/SA (Rs=0.28 and 0.26) and EN/PA (Rs=0.24 and 0.32). M/D and IU were as expected negatively correlated to maltreatment scales with stronger correlations for the full-length IU scale than for M/D. Here, the strongest correlations concerned EN, CTQ total, and EA, while SA and PA were uncorrelated or poorly correlated to IU and M/D. Same-scale correlations over time (W3/W5) were strong (Rs>0.40) for M/D, IU, EA, CTQ total, and EN, but more moderate on SA, PN and PA.

The possible impact of Minimisation/Denial on these diagonal correlations was studied by repeating the W3/W5 correlations with separated analyses for those with and without indications on M/D. Excluding all cases with any M/D-score increased W3-W5 correlations for the total CTQ and three of the five subscales (PN, EA, SA), with the opposite pattern of decreased correlations for the group that had some positive M/D-scores (CTQ total, EN, PN, EA, SA). However, PA showed changes in the opposite direction, with increased correlation for those with M/D scores and decreased correlation for those without. It should be noted that the n of those with positive M/D scores was close to twice the n of those without.

### Internal consistencies

Internal (inter-item) consistencies of all full-length scales – not M/D – are presented in Table 3.

**Table 3:** *Internal consistencies (Cronbach’s alpha) of the CTQ and its subscales*

	Community sample				Clinical Sample
	Wave 2	Wave 3	Wave 4	Wave 5	
n=	1466	1277	721	950	74
CTQ total	-	0.88	0.89	0.80	0.93
EN	0.83	0.85	0.87	0.88	0.90
PN	-	0.39	0.43	0.33	0.75
EA	0.70	0.69	0.75	0.71	0.91
PA	-	0.79	0.79	0.79	0.78
SA	-	0.91	0.94	0.88	0.96
IU (a)	0.44	0.60	0.64	0.69	-

a) IU could not be calculated for the clinical sample, since only dichotomised replies on the MD items were recorded

CTQ=Childhood Trauma Questionnaire, EN=Emotional Neglect, PN=Physical Neglect, EA=Emotional Abuse, PA=Physical Abuse, SA=Sexual Abuse, M/D=Minimizing/Denial, IU=Idealizing Upbringing

In all studied waves of the community sample, we found substantial to excellent consistencies for CTQ total (α:s=0.80-0.89), and for four of its subscales – i.e., EN (α:s=0.83-0.88), PA (all α:s=0.79), SA (α:s=0.88-0.94), and EA (α:s=0.70-0.75; although tangible to substantial in Wave 3, α = 0.69). The three items forming M/D were examined in full length, as the scale named IU, and then showed acceptable internal consistency (in three of four waves (α:s=0.60-0.69), but poor in Wave 2 (α = 0.44). The most problematic scale was PN with moderate internal consistencies in all three waves (α:s=0.33-0.43). In the clinical sample, however, internal consistencies were even better, substantial for PN and PA (α:s=0.75 and 0.78), and excellent for all other scales (α:s=0.90-0.96).

### Test-retest consistencies

In Table 4, we analysed one-year test-retest consistencies between pairs of waves among the three waves with identical timeframes. The analyses were conducted on item level by systematic i.e., symmetric correlation (γ), shown in the first part of the table. Below at the bottom of the table, the mean item consistencies for all scales are given. In the second part of the table the directionality of inconsistencies is analysed.

**Table 4:** *One-year test-retest consistencies of items and scales concerning CTQ subscales, tested with symmetric correlation, i.e., Gamma (γ). Inconsistencies are analysed for possible significant directionality with the Wilcoxon Sign Rank Test*

		Test-retest consistencies (γ)		Test of inconsistencies being directed (a)			
		W2/W3	W3/W4	W2/W3		W3/W4	
Item key words	Subscale	1 year	1 year	z	p	z	p
n =		1174	603				
1. Not enough to eat	PN		0.53			-0.96^b^	0.339
2. Someone to take care of and protect me*	PN		0.54			-0.09^b^	0.929
3. Called “stupid”,” lazy”. or “ugly”	EA	0.60	0.66	**-2.82**^**c**^	**0.005**	-0.03^c^	0.976
4. Parents too drunk/high to take care	PN		0.92			-1.34^b^	0.182
5. Someone helped me feel important*	EN	0.62	0.75	-0.21^c^	0.833	-1.54^b^	0.123
6. Had to wear dirty clothes	PN		0.85			-0.03^c^	0.974
7. Felt loved*	EN	0.59	0.75	**-9.66**^**b**^	**0.000**	-0.83^c^	0.405
8. Thought my parents wished I had never been born	EA	0.52	0.69	-1.93^c^	0.054	-1.09^c^	0.275
9. Hit so hard that I had to see a doctor	PA		0.90			-0.02^c^	0.988
10. Nothing I wanted to change in my family	MN	0.26	0.45	**-4.11**^**b**^	**0.000**	-0.60^c^	0.549
11. Hit me so hard that it left bruises or marks	PA		0.75			-0.31^b^	0.756
12. Punished with belt, board, cord or another hard object	PA		0.92			-1.26^b^	0.209
13. My family looked out for each other*	EN	0.74	0.76	-1.38^b^	0.168	**-3.42** ^**c**^	**0.001**
14. My family said hurtful or insulting things to me	EA	0.70	0.75	-1.88^c^	0.060	-1.28^b^	0.200
15. Physically abused	PA		0.81			-1.00^b^	0.317
16. Perfect childhood	MN	0.69	0.74	**-4.19**^**b**^	**0.000**	-1.21^c^	0.227
17. Got hit badly … noticed by teacher, neighbour, or doctor	PA		0.77			-0.93^b^	0.350
18. Someone in my family hated me	EA	0.74	0.79	-0.69^c^	0.490	-0.45^b^	0.654
19. Family felt close to each other*	EN	0.66	0.65	**-2.73**^**c**^	**0.006**	-1.45^c^	0.148
20. Someone tried to touch me in a sexual way or tried to make me touch them.	SA		0.94			-0.66^b^	0.507
21. Threatened to hurt me unless I did something sexual with them	SA		0.85			-1.81^b^	0.070
22. The best family in the world	MN	0.73	0.77	-0.50^c^	0.615	-1.20^c^	0.229
23. Someone tried to make me do sexual things… watch sexual things	SA		0.91			-1.08^b^	0.279
24. Someone molested me	SA		0.93			-0.34^b^	0.731
25. I was emotionally abused	EA	0.53	0.86	**-8.24**^**c**^	**0.000**	**-2.00**^**b**^	**0.045**
26. Someone to take me to the doctor if I needed it*	PN		0.72			-1.81^c^	0.070
27. I was sexually abused	SA		0.91			-0.77^b^	0.441
28. My family gave strength and support *	EN	0.64	0.71	**-4.76**^**b**^	**0.000**	**-2.36**^**c**^	**0.018**
PN mean γ			0.71				
EN mean γ		0.65	0.72				
EA mean γ		0.62	0.75				
PA mean γ			0.83				
SA mean γ			0.91				
IU mean γ		0.57	0.65				

* The item has reversed scoring; (a) Wilcoxon Sign Ranks Test; with significant inconsistencies in bold; (b) Based on negative ranks, i.e. second test gives lower value; (c) Based on positive ranks, i.e. second test gives higher value

We found that of the 13 W2/W3 test-retest consistencies on item level, eight were substantial, four moderate and one only fair, while of the 28 W3/W4 consistencies, 11 were almost perfect, 14 substantial and three were moderate. On scale level (means of item γ), consistencies were substantial for PN, EN and EA and almost perfect for PA and SA. For IU, consistency started as fair (W2/W3) and increased to substantial (W3/W4). All test-retest consistencies increased from W2/W3 to W3/W4.

Still, some item inconsistencies occurred. In the first test-retest (W2/W3), inconsistencies showed significant directionality for seven out of 13 items; five of which (items no. 3 [EA], no. 7 [EN], no. 10 [IU], no. 16 [IU], and no. 19 [EN]) changed to a more positive evaluation of childhood experiences, while two (no. 25 [EA] and 28 [EN]) changed to a more negative evaluation. In the second test-retest (W3/W4), only three items out of 28 (no.13 [EN], no. 25 [EA] and no. 28 [EN]) showed significant directionality, all resulting in more positive evaluations for these items after one year.

Two of the inconsistent items in W2/W3 (no. 10 and no. 16) belong to the M/D scale and its full-length equivalent IU. The changes meant lower scores in W3 than in W2, i.e., less idealising over time for those giving the least consistent replies on these two items. To test the impact of extreme M/D-values (score 5), we repeated the analyses separately for groups based on M/D scores. There were no inconsistencies in those who scored M/D=0, but inconsistencies occurred when M/D=1-2. As for M/D=3, the inconsistencies were about the same, although they did not reach significance due to fewer cases. Thus, inconsistencies seemed to occur more among those who scored at least one or two extreme M/D-values.

### Convergent validity

Self-rated exposure for abuse and neglect and idealisation of childhood should be related to judgments about the family climate, about parental relations as well as about individual emotional health (both positive health and emotional problems) for data collected in the same wave. These relations are shown as correlations in Table 5. Expected positive correlations as well as expected negative correlations are interpreted as convergent validity.

**Table 5:** *Correlations with family climate, parental relations, and emotional health for CTQ scales in community sample, waves 2, 3 and 5, and in clinical sample*

		EN	PN	EA	PA	SA	IU
**Community sample**							
Wave 2	Family cohesion	- 0.64***	--	- 0.47***	--	--	0.50***
	Family conflict	0.49***	--	0.48***	--	--	- 0.41***
	Psychological health	- 0.47***	--	- 0.38***	--	--	0.43***
	SDQ Emotional problems	0.27***	--	0.27***	--	--	- 0.28***
	SDQ Problem impact	0.33***	--	0.33***	--	--	- 0.29***
Wave 3	Psychological health	- 0.47***	- 0.29***	- 0.36***	- 0.10***	- 0.09**	0.43***
	MHC-SF	- 0.47***	- 0.29***	- 0.33***	-0.15 ***	- 0.11***	0.43***
	SDQ Emotional problems	0.31***	0.18***	0.34***	0.08**	0.06*	- 0.30***
	SDQ Problem impact	0.30***	0.16***	0.29***	0.05	0.02	- 0.29***
	Witnessed domestic violence	0.32***	0.31***	0.37***	0.40***	0.31***	- 0.25***
Wave 5	Family cohesion	- 0.62***	- 0.28***	- 0.44***	- 0.19***	- 0.12***	0.51***
	Family conflict	0.39***	0.18***	0.42***	0.21***	0.13***	- 0.40***
	Perceived maternal support	- 0.60***	- 0.27***	- 0.40***	- 0.12***	- 0.06	0.45***
	Perceived paternal support	- 0.52***	- 0.25***	- 0.41***	- 0.16***	- 0.01	0.44***
	Parental substance misuse	0.21***	0.32***	0.26***	0.24***	0.22***	- 0.29***
	Witnessed domestic violence	0.27***	0.25***	0.31***	0.41***	0.25***	- 0.29***
	Psychological health	- 0.38***	- 0.16***	- 0.35***	- 0.17***	- 0.14***	0.38***
	MHC-SF	- 0.43***	- 0.21***	- 0.32***	- 0.19***	- 0.17***	0.36***
**Clinical sample**							
	Witnessed domestic violence (a)	0.36 **	0.36 **	0.50***	0.52***	0.47***	-
	MHC-SF (a)	-0.35**	-0.14	-0.33**	-0.32**	-10	-

a) IU could not be calculated for the clinical sample, since only dichotomised replies on the M/D items were recorded.

Family climate: *Family cohesion* and *family conflict* were examined in Wave 2 and three to four years later in Wave 5 of the community sample. On both occasions, cohesion was strongly negatively related to EN and EA, and positively related to IU, while conflict was correlated to the same subscales in opposite directions, as could be expected since they concerned positive vs. negative aspects of family climate. All these correlations were strikingly similar after three to four years. In Wave 5, moderate correlations in the same directions were also found concerning PN, PA, and SA. Thus, relations to family climate factors were consistent over the years and correlations were in the expected directions and varied between subscales in expected ways. *Being witness to domestic violence* was included in waves 3 and 5, and with similar moderately strong relations to all scales, with a somewhat stronger correlation to physical abuse, also related to violent behaviour in the family.

Parental relations: *Perceived maternal and paternal support* were examined only in Wave 5, with correlations in the expected pattern as described above for family climate, except for non-relation to SA. Parental substance misuse was only included in Wave 5 and found to be moderately correlated to all subscales in expected directions, with a somewhat stronger correlation to PN, which could be expected since that subscale included item 4, i.e., parents being too drunk to take care of the family.

Emotional health: *Psychological health* was included in all waves and showed a very similar pattern over the years, although with somewhat weaker correlations in late adolescence. For all years, the strongest correlations were negative to EN and EA, and positive to IU. Correlations to PN, PA and SA were more moderate. The *Mental Health Continuum (MHC-SF)* showed a similar consistent pattern in waves 3 and 5 of the community sample, with the strongest negative correlations to EN and EA, and a positive correlation to IU. In the clinical sample, these correlations tended to be in the same direction (although IU could not be studied), but with a stronger negative correlation to PA. The correlations with PN and SA were weaker here too, and with the lower number of subjects in this sample, they did not reach statistical significance. Emotional problems and the impact of problems are two subscales in the *Strength and Difficulties Questionnaire*. They were included in community sample waves 2 and 3 with very similar, moderately strong correlations to EN, EA and IU, and weak correlations (or none) to the other scales.

### Discriminant ability

All 25 items of the CTQ problem subscales were used in a stepwise discriminant analysis between the clinical population (n=74) and the LoRDIA-population (n[W5]=901). Wave 5 of the LoRDIA population was used to create more similar age groups (means of 18 and 17 years, respectively). Although the discriminant analyses managed to correctly classify 93.3% of all persons, the discriminant ability does not seem to be impressive, due to an imbalance in the samples between the proportion of those correctly classified. In the larger LoRDIA population, nearly all (96.8%) were correctly classified, but in the clinical population only slightly more than half were (51.4%). However, that is better than it seems, given that the by-chance classification of the clinical group would have been 7.6%, i.e., the discriminant function provided an improvement of 6.8 times. Nine items accounted for the discriminant function: item 3 from EA; items 4, 13 and 19 from EN; item 6 from PN; items 9, 15 and 17 from PA; and item 27 from SA. Thus, the items forming the discriminant function are distributed on all subscales, i.e., they all contribute. Separating genders did not improve the discrimination.

### Percentiles and categories of severity

The percentiles can be used for comparisons to interpret assessment of individuals in clinical work. These are presented here in Table 6, separately for boys and girls, for two different adolescent age groups of the community sample, i.e., 14+ and 17 years, and for the clinical sample aged 14-25 years, with a mean age of 18.

**Table 6:** *Raw scores and percentiles of CTQ maltreatment subscales for boys and girls in a community sample, aged 14+ and 17 and for a clinical sample, aged 14-25 (mean 18 years). Severity categories above minimal are marked low+, moderate++, severe+++*

		EN		PN		EA	PA	SA
		Boys in community sample, 14+ years old (Wave 3); n = 645						
Percentiles	10	5		5		5	5	5
	50	6		5		6	5	5
	60	7		6		6	5	5
	70	8		6		7	5	5
	80	9		9*		9*	5	5
	90	12*		9*		10*	7	5
	95	15**		11**		12*	8*	5
	96	15**		12**		13**	9*	6*
	97	17**		13***		13**	10**	8**
	98	18***		13***		14**	13***	13***
	99	20***		15***		18***	17***	15***
		Boys in community sample, 17 years old (Wave 5); n = 415						
Percentiles	10	5		5		5	5	5
	50	6		5		5	5	5
	60	7		6		6	5	5
	70	9		6		7	5	5
	80	10*		8*		8	5	5
	90	12*		9*		10*	6	5
	95	14*		11**		12*	7	5
	96	15**		11**		12*	8*	5
	97	17**		12**		13**	9*	6*
	98	17**		13***		14**	10**	7*
	99	23***		14***		17***	15***	14***
		Boys in clinical sample, 14-25 years old (mean 18); n = 45						
Percentiles	10	5		5		5	5	5
	50	8		6		7	5	5
	60	10*		6		8	5	5
	70	12*		8*		8	5	5
	80	13*		11**		10*	6	5
	90	17**		14***		13**	7	6*
	95	20***		17***		18***	10**	12**
	96	21***		17***		19***	11**	15***
	97	23***		18***		21***	12**	18***
		Girls in community sample, 14+ years old (Wave 3); n = 659						
Percentiles	10	5	5		5		5	5
	50	6	5		6		5	5
	60	7	5		6		5	5
	70	8	6		7		5	5
	80	9	7		9*		5	5
	90	12*	9*		11*		6	5
	95	15**	10**		13**		8*	5
	96	15**	10**		13**		8*	5
	97	16**	11**		14**		9*	5
	98	17**	12**		16***		10**	7*
	99	19***	13***		18***		11**	12*
		Girls in community sample, 17 years old (Wave 5); n =520						
Percentiles	10	5	5		5		5	5
	50	6	5		6		5	5
	60	6	5		6		5	5
	70	8	6		7		5	5
	80	9	7		8		5	5
	90	11*	9*		10*		5	5
	95	14*	10**		13**		6	6*
	96	14*	10**		13**		7	7*
	97	15**	11**		15**		8*	7*
	98	16**	11**		15**		9*	8**
	99	18***	13***		18***		10**	12**
		Girls in clinical sample, 14-25 years old (mean 18); n = 29						
Percentiles	10	5	5		5		5	5
	50	9	7		8		5	5
	60	11*	8*		12*		5	5
	70	12*	9*		13**		6	5
	80	14*	10**		15**		8*	9**
	90	19***	15***		22***		12**	19***
	95	20***	18***		24***		17***	24***
	96	20***	19***		24***		18***	24***

Based on ROC curve estimation of sensitivity and specificity for each subscale against criteria measures from Evaluation of Life-time Stressors, Bernstein and Fink (1998, 2011) proposed cut-offs to create categories of severity: None or minimal (EA: 5-8; PA: 5-7; SA: 5; EN: 5-9; PN: 5-7); Low (EA: 9-12; PA: 8-9; SA: 6-7; EN: 10-14; PN: 8-9); Moderate (EA: 13-15; PA: 10-12; SA: 8-12; EN: 15-17; PN: 10-12) and Severe (EA: ≥16; PA: ≥13; SA: ≥13; EN: ≥18; PN: ≥13). These categories are visualised by colours in the table.

## Discussion

This study contributes to a growing body of evidence showing the CTQ to be an acceptable and reliable instrument to be used in both clinical screening and research on child maltreatment. In addition, our study provides new knowledge on the validity and reliability of the use of the CTQ in adolescents.

The CTQ was found to be well accepted among respondents in early and late adolescence from a clinically naïve community sample. Previously, it was found to be accepted in an adult and severely traumatised group with many clinical experiences (Lundgren et al., 2002). Thus, the acceptability of the CTQ has been shown in very different groups.

The means of the maltreatment scales (CTQ total and five subscales) in the community sample showed small differences over time, and as expected, the clinical sample scored higher than the community sample on all scales except for the M/D. Maltreatment scales were highly or moderately positively correlated within waves, while same-scale correlations over 2-3 years (W3/W5) were strong for most scales and moderate on others.

In the community sample, internal consistencies were substantial to excellent for the CTQ total and four of its subscales and from early to late adolescence. The exception was PN, with only moderate internal consistency. The additional IU scale showed acceptable internal consistency in three of four waves. In the clinical sample, internal consistencies were even better and substantial also for PN. The latter finding was not expected since most other studies report lower consistency for PN (e.g., Gerdner & Allgulander, 2009; Karos et al., 2014; Paivio & Cramer, 2004; Sölva et al., 2020).

Test-retest consistencies *on item level* varied from fair to substantial in W2/W3 and from moderate to almost perfect in W3/W4. Mean one-year test-retest consistencies *of scales* were substantial or almost perfect, and for all scales, they increased from early to mid-adolescence. As mentioned above, Hardt and Rutter (2004) claim that comparisons of contemporaneous and retrospective accounts obtained in epidemiological/longitudinal studies of non-clinical populations are among the best methods to assess biases in reporting, i.e., invalidity. The same approach was used by Fan et al. (2006) who recommends repeated longitudinal measures to assess validity of behaviours that are unlikely to be externally validated. After one year, it would in general be impossible to remember replies to specific questions, not knowing that they would be repeated in coming data collections. We argue therefore that substantial stability in long-term test-retest adds support to the validity of the instrument. Furthermore, since test-retest stability increased over time on item as well as scale level despite the longer recollection time, this would indicate that adolescents provide more credible and reliable answers from the age of 14 than before.

Previous test-retest studies on the CTQ-SF have been conducted on adult populations (e.g., Paivio & Cramer, 2004) showing acceptable consistency. Our findings, however, contribute to a growing body of evidence on test-retest consistency in three aspects. First, our test-retest data have a longer follow-up time than most, approximately one year in between. This is only outnumbered by Shannon et al. (2016) who administered the CTQ with 18 months between the times. Second, our test-retest studies are on adolescents, which to our knowledge has not been studied before. Third, starting the analyses on item level, using systematic correlation, and combining with analyses of inconsistencies have not previously been done.

A closer look at the directionality of inconsistencies can give us more understanding. Non-directed inconsistencies indicate that the problem concerns lack of precision (i.e., a problem of reliability), while directed inconsistencies indicate systematic bias (i.e., a problem of validity). Although about 10% of items in the W3/W4 test-retest showed significant directionality, this is a sharp decrease from W2/W3 where more than half of tested items showed directed inconsistencies. The retrospective test therefore seems more valid when conducted at the age of 14-15 than at earlier ages.

Convergent validity was tried in relation to scales on family climate, parental relations, and emotional health. All correlations were in the expected directions, and they varied in expected ways between subscales. The stronger correlation between physical abuse and being witness to domestic violence can serve as one example. This is not surprising since previous research found that physical abuse is common among children witnessing domestic violence (Broberg et al., 2011). Other examples are the expected stronger relation between physical neglect and parental substance misuse, and the expected findings concerning emotional neglect and abuse having a strong relation to all scales on emotional health. The findings thereby provide support for convergent validity. They also showed consistent patterns over time, also from the earliest age.

The discriminant ability was, perhaps, not as good as expected. The CTQ was able to correctly classify almost all respondents in the community sample. Although only half of the respondents in the clinical sample were correctly classified, this was almost seven times better than by-chance. Therefore, we cannot conclude that it discredits the validity of the CTQ.

Means and norm data from the clinical sample and the community sample showed that the clinical sample scored higher on all scales. This is expected since child maltreatment has been robustly associated with problematic alcohol/substance use in adolescence (e.g., Alvarez-Alonso et al., 2016; Hagborg et al., 2020). Norm data are presented for clinical guidance with options to choose between reference groups.

The findings show that the CTQ-SF is acceptable to adolescents in a community population, and that it is valid and reliable for use in adolescent populations. Our findings are in line with previous studies showing that adolescents generally report meaningful and positive experiences of answering questions about child maltreatment (e.g., Chu et al., 2008; Hasking et al., 2015).

### Implications concerning early adolescents

The long-term stability in measurement and the convergent validity led us to the conclusion that the adolescents provided valid responses. The findings concerning discrimination ability do not discredit that. However, some caution is advised when applied to early adolescents aged 12-13 years, since the test-retest stability is then weaker.

Lastly, we can evaluate the M/D scale when used in adolescents. The interpretation of the scale signalising caution for having bias is supported by the fact that *same-scale correlations* (Table 2) increased when those with positive M/D scores were excluded. In addition, there were no test-retest *item inconsistencies* when they were removed. Most adolescents, however, had some positive M/D scores (Table 2) and removing them altogether would harm the inference of the study even more. A further look at the inconsistency analyses (Table 5) show that the scale mostly affected was the M/D scale, with 2 out of 3 items having significantly directed inconsistencies. This finding stresses the need for some caution when interpreting the M/D scale in early adolescence, since it is the one with most unstable measurement.

*In conclusion*. The CTQ has acceptability when used in adolescence and can be trusted to give consistent and valid self-reports on childhood maltreatment that occurred before the age of 12. However, some caution is advised when the CTQ is used in early adolescence up to the age of 14 years, and when interpreting the M/D scale at such a young age.
